# Novel application of neural network modelling for multicomponent herbal medicine optimization

**DOI:** 10.1038/s41598-019-51956-6

**Published:** 2019-10-28

**Authors:** Yong-Shen Ren, Lei Lei, Xin Deng, Yao Zheng, Yan Li, Jun Li, Zhi-Nan Mei

**Affiliations:** School of Pharmaceutical Science, South-Central University for Nationalities, Wuhan, Hubei China

**Keywords:** Translational research, Bioanalytical chemistry, Virtual screening

## Abstract

The conventional method for effective or toxic chemical substance identification of multicomponent herbal medicine is based on single component separation, which is time-consuming, labor intensive, inefficient, and neglects the interaction and integrity among the components; therefore, it is necessary to find an alternative routine to evaluate the components more efficiently and scientifically. In this study, sodium aescinate injection (SAI), obtained from different manufacturers and prepared as “components knockout” samples, was chosen as the case study. The chemical fingerprints of SAI were obtained by high-performance liquid chromatography to provide the chemical information. The effectiveness and irritation of each sample were evaluated using anti-inflammatory and irritation tests, and then “Gray correlation” analysis (GCA) was applied to rank the effectiveness and irritability of each component to provide a preliminary judgment for product optimization. The prediction model of the proportions of the expected components was constructed using the artificial neural network. The results of the GCA showed that the irritation sorting of each SAI component was in the order of B > A > G > J > I > H > D > F > E > C and the effectiveness sorting of SAI components was in the order of D > C > B > A > F > E > H > I > G > J; the predictive proportion of SAI was optimized by the BP neural network as A: B: C: D: E: F = 0.7526: 0.5005: 5.4565: 1.4149: 0.8113: 1.0642. This study provided a scientific, accurate, reliable, and efficient approach for the proportion optimization of multicomponent drugs, which has a good prospect of popularization and application in product upgrading and development of herbal medicine.

## Introduction

The horse chestnut seed is a commonly used herbal medicine and has been obtained from several species of *Aesculus*, including the European horse chestnut (*Aesculus hippocastanum* L.), Japanese horse chestnut (*A. turbinata* Blume), Qiyeshu (*A. chinensis* Bge), and Tianshisu (*A. wilsonii* Rehd)^1–3^. In Europe and America, the seeds have usually been used to treat venous disorders, particularly varicose veins and hemorrhoids, as well as inflammatory ailments such as arthritis, backache, strains, tendonitis, and sports injuries^4^. In Asia, the seeds have been utilized for remedying sore throats and eye diseases, passing kidney stones and relieving stomach ache, alleviating hemorrhoids symptoms, and so on^5,6^.

Various components such as saponins, flavonoids, coumarins, organic acids, and sterol compounds have been separated and identified from this genus^7^. Of these, over 30 pentacyclic triterpenoid classes of saponins have been the most popularly focused chemical constituents of the horse chestnut, including more than 10 aescins.

**Figure 1:**
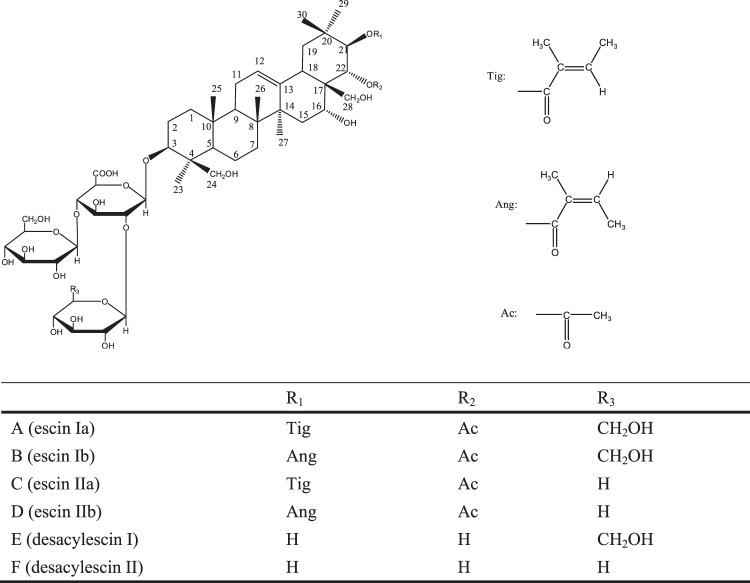
Chemical structures of A (escin Ia), B (escin Ib), C (escin IIa), D (escin IIb), E (desacylescin I) and F (desacylescin II).

Aescin is the dominant effective ingredient of horse chestmut saponins, based on its chemical structure, and can be divided into α, β, and krypto aescins. The most characteristic component of aescins A and B is β-escin, for aescins C and D it is α-escin, and there are isomers (C_55_H_86_O_24_) with different positions of the acetyl group, which might notably affect potency and toxicity^8^. The chemical structure of aescin A, B, C, D, E, and F are shown in Fig. 1.

Previous studies have shown the anti-inflammatory, anti-exudation, anti-edema, and anti-tumor effects, as well as the reduction in vascular permeability, inhibition of lysosome activity, an increase of vascular tension, and improvement in the microcirculation effects by aescin^9^. However, the poor water solubility of aescin has led to low bioavailability. This issue is currently managed by converting aescin into sodium aescinate (SA) for clinical application.

There are different preparations of SA for clinical application worldwide, which are adapted to different clinical needs, e.g., Repanl (injection) and Qescusan, Germd, and Madaus (gel, sugar coated tablets, and suppositories, respectively) in Germany; Venostascin (injection, capsule, and ointment) and Tochikinon (tablet) in Japan; and the main application forms in China include SA injection (SAI), SA tablets, and gels^10^. The main clinical uses of SA are for cerebral edema, trauma, tenosynovitis, internally soft tissue injury, postoperative swelling, superficial thrombotic phlebitis, varicosity, venous drainage disturbance, and intravenous nursing after administration^11^. SAI has been extensively used for treating acute and critical diseases such as encephaledema^12^, owing to its definite curative effect. Unfortunately, there have been some adverse drug reactions^13^, mainly vascular irritation at the injection site, anaphylaxis, and even allergic shock and renal or hepatic injuries, which have restricted its popularization. The main cause of SAI irritations is the drug itself; however, the irritation mechanism of SAI and how to avert it remains unclear.

Being an effective fraction herbal medicine, many similar chemical ingredients are contained in SAI, such as SA A, B, C, D, E, and F^14^. Generally, SA A, B, C, and D are the primary effective ingredients, and their proportions might affect the effectiveness and security of SAI. However, the effectiveness and/or irritation contribution of the primary components of SA A, B, C, and D, and their ideal ratios remain unclear. Additionally, the role of the lower content ingredients, i.e., SA E, F, and other trace components in SAI have undergone limited studies.

To solve the above issues and relieve the suffering of patients from adverse drug reaction and improve clinical efficacy, a novel strategy for irritant component identification and ingredient optimization was proposed in the present study as follows: “components knockout” technology. “Components knockout” technology is taken reference to the molecular biology method and mode of “gene knock-out” or “gene deletion” with some different^15,16^. In this study, we separated the individual components of the SAI by preparative liquid chromatography. Secondly, the remaining components were analyzed by high performance liquid chromatography (HPLC). Thirdly, the main pharmacodynamics effects and irritancy were assessed by anti-inflammatory and stimulation tests; apply Gray correlation analysis (GCA) to rank the effectiveness and irritability of each component, and use the artificial neural network (ANN) to optimize the proportion of each component to obtain the recommended composition and ratio of SAI, and provide a technique for product upgrading of multicomponent herbal medicine.

**Figure 2:**
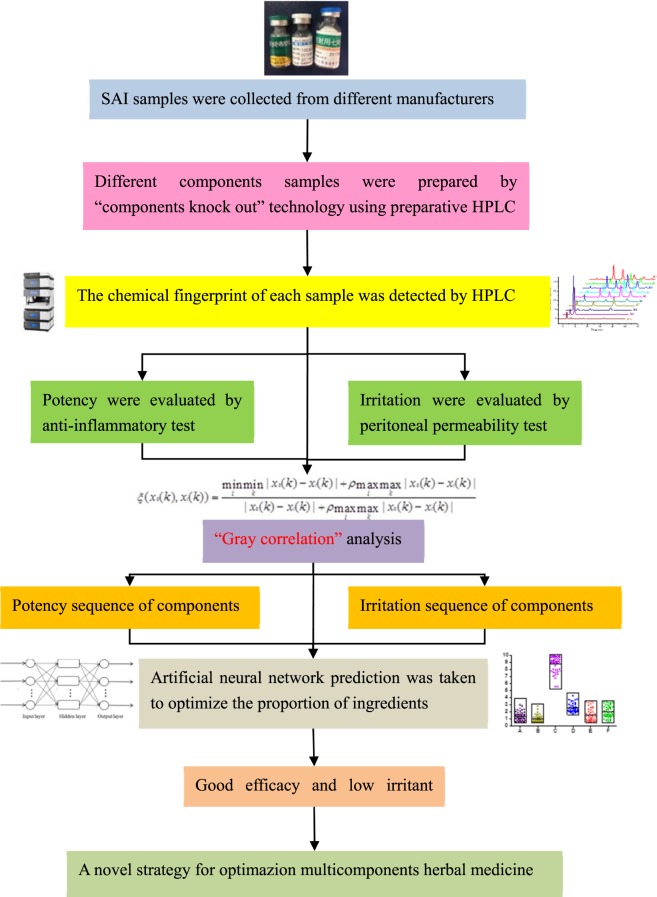
The overall technology flowchart.

The overall technology flowchart is shown in Fig. 2.

## Results

### Irritation test

**Figure 3:**
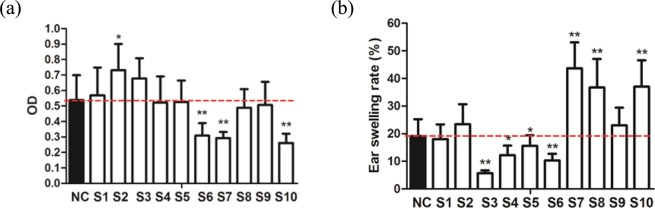
(**a**) SAI induced peritoneal capillary permeability on mice (Irritation test). NC (normal control group), S1 (Standard), S2 (CHHU), S3 (BALU), S4 (PUYT), S5 (PUYT), S6 to S10 (Separation from CHHU). All data were expressed as means ± SEM (n = 8). *p < 0.05 compared to the normal control group and **p < 0.01compared to the normal control group. (**b**) Efficacy of SAI on xylene induced ear swelling on mice (Anti-inflammatory efficacy test). NC (normal control group), S1 (Standard), S2 (CHHU), S3 (BALU), S4 (PUYT), S5 (PUYT), S6 to S10 (Separation from CHHU). All data were expressed as means ± SEM (n = 8). *p < 0.05 compared to the normal control group and **p < 0.01 compared to the normal control group.

As shown in Fig. 3(a), the ODs of samples S1, S2, and S3 were significantly higher than that of the NC group, especially for sample S2. The ODs of the other samples were lower than that of the NC group, especially for samples S6, S7, and S10 (p < 0.01). Therefore, samples S1, S2, and S3 might have more obvious leakage of aescin, and thus more harmful irritation; conversely, the leakage of aescin in samples S6, S7, and S10 was lower, indicating less irritation caused by these samples on mice.

### Anti-inflammatory efficacy test

As shown in Fig. 3(b), the ear swelling ratios of samples S3, S4, S5, and S6 were significantly lower than that of the NC group (p < 0.05 or p < 0.01), indicating inhibitory effects on xylene-induced mouse ear swelling. The ear swelling ratios of samples S2, S9, S7, S8, and S10 (p < 0.01) were higher than that of the NC group, indicating aggravation effects on xylene-induced mouse ear swelling.

### GCA

Based on the “Gray correlation” theory, the higher the value of the GCD, the closer the sequence of associated factors is to the behavioral characteristics. Table 1 shows that the irritation sorting of each SAI component was in the order of B > A > G > J > I > H > D > F > E > C, indicating that aescins B and A had higher irritation than that of aescins D, E, C, and F. Table 2 shows that the effectiveness sorting of SAI components was in the order of D > C > B > A > F > E > H > I > G > J, indicating that aescins D and C were more potent than that of aescins B, A, F, and E. The potency of G, H, I, and J were lower than that of the other components; nevertheless, their irritations were in the middle of the sequence. Considering their low abundance, these ingredients should be eliminated from SAIs.

**Table 1:** Gray correlation analysis of each sample peak area with efficacy.

Peak number	Gray correlation grade	Sequence
A	0.8670	2
B	0.8804	1
C	0.7355	10
D	0.7882	7
E	0.7451	9
F	0.7692	8
G	0.8131	3
H	0.8043	6
I	0.8076	5
J	0.8128	4

**Table 2:** Gray correlation analysis of each sample peak area with irritation.

Peak number	Gray correlation grade	Sequence
A	0.7788	7
B	0.7665	8
C	0.7232	9
D	0.6982	10
E	0.8029	5
F	0.7902	6
G	0.8135	2
H	0.8032	4
I	0.8075	3
J	0.8152	1

Thus, to enhance the efficacy of SAI and decrease its irritation, the component proportion of SAI should be optimized by increasing the content of aescins D and C and decreasing the content of aescins B and A, and thus the content of aescins E and F should be improved appropriately.

### ANN prediction

#### ANN construct

**Figure 4:**
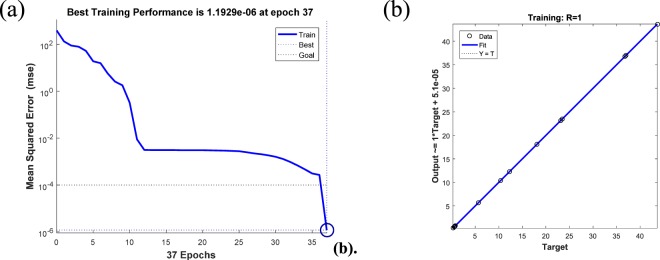
(**a**) Neural network data training chart. **(b**) Neural network regression model correlation.

The BP neural network was trained by nine set samples, and the results showed that with an increase of training iterations, the neural network fitting error decreased. As shown in Fig. 4(a,b), after training the model error reached 10^−6^, the model determination coefficient R value was as high as 1, the fitting curve coincided with the target curve, and the linear regression effect was obtained between the output result and the target result.

The established network was verified by the reserved sample (S5) and the predictive output was 0.519 and 27.289, which was very close to the experimental result (0.525 and 27.559), indicating that the neural network was accurate and reliable.

#### The proportion optimization results of the BP neural network

Based on the results of the GCA and the original proportion of ingredients of the SAI products, the expected proportion range of components was set as A: 0.5–4, B: 0.5–3, C: 5–10, D: 1.5–6.5, E: 0.5–3.5, and F: 0.5–3.5, which decreased the proportion of SA A and B, and increased the proportion of SA C, D, E, and F.

**Figure 5:**
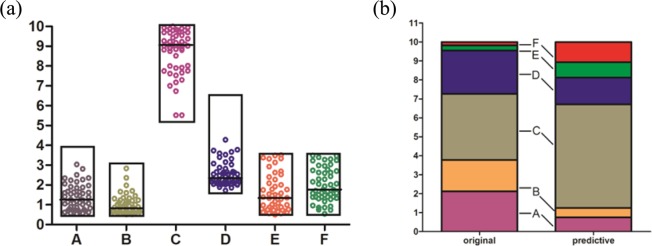
(**a**) 50 times proportion predication distribution diagram. According to the results of “Grey correlation” analysis and the original ingredients proportion of SAI products, the expected proportion range of components was set as A: 0.5–4, B: 0.5–3, C: 5–10, D: 1.5–6.5, E: 0.5–3.5, F: 0.5–3.5 respectively, and 50 groups data which meeting the criterion (absorbance <0.539, ear swelling rate <19.198%) was collected. (**b**) Original and predictive proportion comparation of components in SAI. Compare with the original proportion, the proportion of sodium aescinate C (gary), E (green), F (red) was remarkably enhanced, and the proportion of A (purple), B (orange), D (blue) was decreased accordingly.

A total of 50 groups of data that met the criteria (absorbance <0.539 and ear swelling rate <19.198%) were collected, and the results are shown in Fig. 5(a). The proportion of the ingredients trended to a relatively concentrated range, indicating that the predicated proportions inclined to stable and the mean value reflected the motion tendency of the component proportions.

Importing the median proportion of the 50 groups (A: B: C: D: E: F = 1.2500: 0.8313: 9.0625: 2.3500: 1.3475: 1.7675) into the BP neural network and setting the absorbance and ear swelling ratio output value to 0.369 and 4.131, respectively, which was close to the median of the 50 predictive groups (0.371 and 4.101) and lower than that of commercial samples, showed that this proportion was more optimal than that of the original samples that had higher potency and lower irritation.

The optimized proportion of SAI components (A: B: C: D: E: F = 1.2500: 0.8313: 9.0625: 2.3500: 1.3475: 1.7675) was then converted into a 10 point proportion as A: B: C: D: E: F = 0.7526: 0.5005: 5.4565: 1.4149: 0.8113: 1.0642, and then compared to the original proportion (A: B: C: D: E: F = 2.1192: 1.6576: 3.4811: 2.2931: 0.2653: 0.1837) of the commercial samples (S1–S5). The results (Fig. 5(b)) showed that the proportions of C, E, and F were remarkably enhanced, and the proportions of A, B, and D were decreased, which showed the probable methods and goals for upgrading the SAI products.

#### Verification of the proportion optimization result

**Figure 6:**
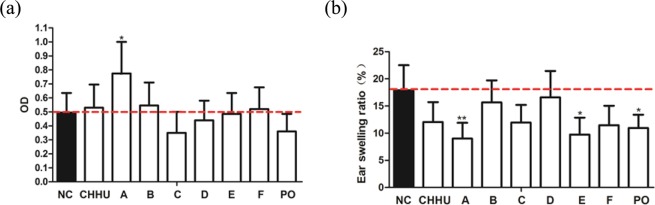
(**a**) SAI induced peritoneal capillary permeability on mice (Irritation test). NC (normal control group), CHHU (CHHU group), A (aescinate A group), B (aescinate B group), C (aescinate C group), D (aescinate D group), E (aescinate E group), F (aescinate F group), PO (proportion optimization group). All data were expressed as means ± SEM (n = 8). *p < 0.05 compared to the normal control group. (**b**) SAI induced peritoneal capillary permeability on mice (Irritation test). NC (normal control group), CHHU (CHHU group), A (aescinate A group), B (aescinate B group), C (aescinate C group), D (aescinate D group), E (aescinate E group), F (aescinate F group), PO (proportion optimization group). All data were expressed as means ± SEM (n = 8). *p < 0.05 compared to the normal control group and **p < 0.01 compared to the normal control group.

As shown in Fig. 6(a,b), the predicted proportion of SAI had lower irritation and better effectiveness.

## Discussion

There are many ingredients in herbal medicine, with the effective or toxic chemical components of most of these remaining unclear. Nowadays, the shortage of single component separation and evaluation for drug action identification methods has been recognized and emphasized. The conventional method for determining effective components is time-consuming, labor intensive, inefficient, and neglects the interaction and integrity among components, which might play a very important role in the pharmacological action of herbal medicine. Therefore, it was necessary to determine an alternative routine to distinguish the primary effective materials of multicomponent drugs more efficiently and scientifically.

GRA is based on the theory of “difference is information,” and compares the differences among known or accessible information (white box) to show the difference of the unknown information (black box), and obtain the useful gray information (the sequence of the GCD) to reveal the black box gradually, which provides a practical operation for understanding the complicated world^17–19^. Multicomponent herbal medicine is believed to be a complex black box, especially traditional Chinese medicine, and its traditional methodology “Observing the Internal to Conjecture the External Pathogenic Factors” was similar to the concept of GRA. This provides a feasible way for the identification and sequencing of the effectiveness and safety of components in herbal medicine.

Artificial intelligence has been one of the major developments in modern science, and its application in the pharmaceutical field provides a powerful prediction method for the development, optimization, and evaluation of new drugs. ANN is a mature artificial intelligence prediction method with high reliability and accuracy. By analyzing the input and output layers to construct a suitable mathematical model, the proportion of components or the prescription of drugs can be optimized^20^.

SAI is an important herbal extract injection for the treatment of acute and severe cerebral edema that is used worldwide. However, its severe vascular irritation and other adverse reactions have constrained its advancement in use. The proportion of ingredients in SAI has a significant influence on its effectiveness and safety. Normally, the main ingredients (A, B, C, and D) account for more than 90% of the total components; however, the appropriate proportion of the components is indeterminate and the proportion varies with different manufacturers, which has caused great inconvenience and hazards for quality control and the clinical use of these injections. Therefore, it was necessary to perform a novel study to identify the effectiveness and safety of these ingredients and optimize their proportion, and thus improve the property and quality of the product.

In the present study, SAI products were obtained from different manufacturers and their chemical fingerprints were analyzed. This showed that there was a great difference in the chemical composition and proportion of the products from each manufacturer. In combination with the clinical use of drugs, there were obvious differences in their effectiveness and safety, which brings greater risks to clinical medication. Several SAI samples were prepared by components knockout technology and the chemical fingerprints of each sample were obtained by HPLC to provide rich comparison samples for the identification of the effectiveness and safety of various components. The results obtained were consistent with those reported in the literature, which provided a preliminary judgment for product optimization, and a prediction model was constructed using the BP neural network, and the expected proportion of each component was set on the basis of GCA. The proportion distribution range of each component was obtained by multiple predictions, the mean value was selected as the best proportion, and verification was performed. The best proportion of each SAI component was found to be A: B: C: D: E: F = 0.7526: 0.5005: 5.4565: 1.4149: 0.8113: 1.0642.

In summary, the present study provided a scientific, accurate, reliable, and efficient approach for the optimization of the proportion of multicomponent drugs based on a combination of component separation and GCA with ANN, which might have a good prospect of popularization and application in product upgrading and development of herbal medicines.

## Materials and Methods

### Chemicals and reagents

Five commercial SAI samples (S1–S5) were obtained from three manufacturers in China. Samples S6–S10 were then isolated from CHHU (S2) using the components knockout technology method and preparative HPLC, based on the method described in sub-section 4.4.1 (The collected SAI samples were shown in Table 3).

**Table 3:** SAI samples collected from different manufacturers.

Brand	Manufacturer	Specification	Sample ID	Lot No.
Standard	China Institute of Food and Drug Control	100 mg	S1	10346-200402
CHHU	HNYG	10 mg	S2	20160303-4
BALU	WHCL	10 mg	S3	20170101 P
PUYT	WHPS	5 mg	S4	20160902-2
PUYT	WHPS	10 mg	S5	20150129-2
Prepared	Isolate from CHHU	—	S6–S10	P20160708

Saline was purchased from Wuhan Binhu Shuanghe Pharmaceutical Co., Ltd. (Wuhan, China), lot number H42020475 (1705030804). Evans Blue was purchased from Shanghai Aladdin Biochemical Technology Co., Ltd. (Shanghai, China). Xylene was purchased from Sinopharm Chemical Reagent Co., Ltd (Shanghai, China). All reagents were analytical grade and all solvents used for HPLC analysis were HPLC grade.

### Animals

Male KunMing mice (35–40 g) were purchased from Hubei Provincial Center for Disease Control and Prevention (SYXK (E) 2016–0089; Wuhan, China). They were housed in a 12 h light-dark cycle at 25 ± 2 °C in 30–60% relative humidity. The mice had access to a standard pellet diet and water continuously throughout the experimental period. The present study acquired clearance from the Institutional Animal Ethical Committee of the Committee for the Purpose of Control and Supervision of Experiments on Animals, South-Central University for Nationalities, Wuhan, China, and conformed to the National Institute of Health Guidelines on the Ethical Use of Animals.

### Instruments and apparatus

The following instruments and apparatus were used during the study: refrigerated centrifuge (DL28R, Shanghai, China); analytical balance (Shanghai, China); UV-Vis spectrophotometer (UV-1800PC, Shanghai, China); Waters Prep LC W600 (UV Detector Waters 996, USA); and Dean U-3000 HPLC (vdw3000 UV detector, USA).

### Chemical analysis

#### Chromatographic separations

Chromatographic separations were performed using a BETASIL C_18_ column (5 µm, 250 × 10 mm; Thermo Scientific, USA). The mobile phases A and B were methyl alcohol and water (40:60), respectively. The flow rate was set at 3 mL·min^−1^. The detection wavelength was set at 220 nm. The column was maintained at 30 °C during the experiment.

Chromatographic analysis was performed using an YMC C_18_ column (5 µm, 250 × 4.6 mm; Thermo Scientific). The mobile phases A and B were acetonitrile and 0.1% acetic acid in water (36:64), respectively. The flow rate was set at 1 mL·min^−1^.

All SAI samples were dissolved in ultrapure water and configured as a 1 mg·mL^−1^ solution. The solution was filtered through a 0.46 µm membrane before analysis and separation.

#### HPLC fingerprint

**Figure 7:**
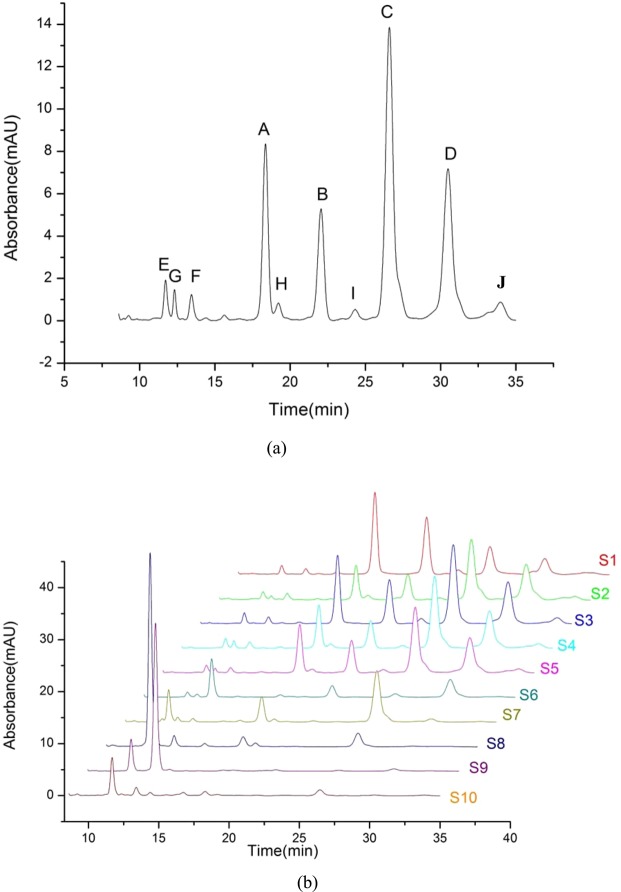
(**a**) HPLC fingerprint for SA. (**b**) Chemical fingerprint of each sample, S1 (Standard), S2 (CHHU), S3 (BALU), S4 (PUYT), S5 (PUYT), S6 to S10 (Separation from CHHU).

The HPLC fingerprint for SA was established and nine chromatographic peaks were identified as SA A, B, C, D, E, F, G, H, and I based on the reported literature (shown in Fig. 7(a)). HPLC fingerprints of each sample were obtained and are shown in Fig. 7(b). The actual peak area and relative peak area of components in each sample were calculated and are shown in Tables 4 and 5, respectively.

**Table 4:** Actual peak area of each component in each sample.

	S1	S2	S3	S4	S5	S6	S7	S8	S9	S10
A	7.2004	2.7394	5.7446	3.5665	4.0388	0.2684	0.2376	0.8617	0.0000	0.3785
B	5.6804	2.3243	4.2589	2.7048	3.1699	1.2157	0.0000	0.0000	0.0000	0.0000
C	3.7543	7.5994	9.3531	9.1671	8.5072	0.4978	6.5026	1.5864	0.0000	0.6717
D	2.5943	5.3110	6.0569	5.7693	5.4610	2.6316	6.5026	0.0000	0.0000	0.0000
E	0.6630	0.4480	0.7114	0.5939	0.5016	0.3686	1.8175	11.4816	1.8821	2.3061
F	0.4603	0.3251	0.4444	0.4252	0.3530	2.5870	2.0621	0.7648	9.5694	0.5752
G	0.0000	0.1446	0.0000	0.4210	0.2148	0.2232	0.2444	0.0000	0.0000	0.0000
H	0.3326	0.0000	0.0000	0.1304	0.0984	0.0000	0.0000	0.0000	0.0000	0.0000
I	0.0000	0.2813	0.0000	0.4488	0.3069	0.0000	0.0000	0.0000	0.0000	0.0000
J	0.5606	0.0642	0.5647	0.3111	0.3666	0.0000	0.0000	0.0000	0.0000	0.0000
Total	21.2459	19.2373	27.1341	23.5383	23.0183	7.7924	17.3667	14.6945	11.4515	3.9315

**Table 5:** Relative peak area of each component in each sample (%).

	S1	S2	S3	S4	S5	S6	S7	S8	S9	S10
A	33.89	14.24	21.17	15.15	17.55	3.44	1.37	5.86	0.00	9.63
B	26.74	12.08	15.70	11.49	13.77	15.60	0.00	0.00	0.00	0.00
C	17.67	39.50	34.47	38.95	36.96	6.39	37.44	10.80	0.00	17.09
D	12.21	27.61	22.32	24.51	23.72	33.77	37.44	0.00	0.00	0.00
E	3.12	2.33	2.62	2.52	2.18	4.73	10.47	78.14	16.44	58.66
F	2.17	1.69	1.64	1.81	1.53	33.20	11.87	5.20	83.56	14.63
G	0.00	0.75	0.00	1.79	0.93	2.86	1.41	0.00	0.00	0.00
H	1.57	0.00	0.00	0.55	0.43	0.00	0.00	0.00	0.00	0.00
I	0.00	1.46	0.00	1.91	1.33	0.00	0.00	0.00	0.00	0.00
J	2.64	0.33	2.08	1.32	1.59	0.00	0.00	0.00	0.00	0.00

### SAI-induced peritoneal capillary permeability on mice (irritation test)

To investigate the irritation of SAI-induced peritoneal capillary permeability on mice, 88 mice were randomly divided into 11 groups: normal control (NC) group and S1–S10 groups for SAI sample S1 to S10. Mice were fasted for 12 h and had free access to water, then the same volume (0.5 mL) of saline and each SAI sample (S1 to S10) was given to each group by intraperitoneal injection; after that, 0.5% Evans Blue (dosage 6.25 mL·kg^−1^) was injected via the tail vein for each group. Mice were sacrificed by vertebral dislocation 2 h later. After injection of 5 mL saline into the peritoneal cavity, the peritoneal fluid was collected in a centrifuge tube. The tube was centrifuged at 3000 × g for 10 min, afterwards the supernatant Optical Density (OD) was measured at 590 nm.

The dosage for SAI groups S1 to S5 was 12.50 mg·kg^−1^, which was 3 times that of a human dose based on the surface area coefficient, and the dosages for S6 to S10 were 8.50, 11.38, 13.45, 4.02, and 5.50 mg·kg^−1^, respectively, based on their total peak area ratio to sample CHHU (S2).

### Efficacy of SAI on xylene-induced ear swelling of mice (anti-inflammatory efficacy test)

To investigate the efficacy of each SAI sample, the xylene-induced ear swellings of the mice were utilized to evaluate the anti-inflammatory efficacy of SAI samples^21^. Another 88 KM mice were used for this experiment, the grouping method and dosage was the same as that for the irritation test (section 4.5). Saline and SAI samples S1 to S10 were injected via the tail vein and, after 30 min, 30 µL xylene was smeared onto the left ear of the mice. After another 30 min, the mice were sacrificed by vertebral dislocation and the two ears were cut out along the baseline of the auricle. Ear biopsies of 8.0 mm in diameter were punched out from the same position and weighed. Then, the weight gain of the ear inflammation and the ear swelling ratio (%) were calculated using the following formulas:

1 $${\rm{Weight}}\,{\rm{gain}}\,{\rm{of}}\,{\rm{inflammation}}={\rm{left}}\,{\rm{ear}}-{\rm{right}}\,{\rm{ear}}$$

2 $${\rm{Ear}}\,{\rm{swelling}}\,{\rm{ratio}}\,( \% )=({\rm{left}}\,{\rm{ear}}-{\rm{right}}\,\mathrm{ear})/{\rm{right}}\,{\rm{ear}}\times 100 \% )$$

### Gray correlation analysis (GCA) method

#### GCA of each sample peak area with efficacy

To assess the actual dosage of each peak (Peaks A to I) in each sample with efficacy (OD) as well as irritation (ear swelling ratio), GCA was utilized as the evaluation system to obtain the sorting by calculating the gray correlation degree (GCD) as follows:

$${\rm{Set}}\,{{\rm{X}}}_{0}=({x}_{0}(1),\,{x}_{0}(2),\ldots ,{x}_{0}({\rm{n}}))\,{\rm{as}}\,{\rm{the}}\,{\rm{sequence}}\,{\rm{of}}\,{\rm{system}}\,{\rm{behavior}}\,{\rm{characteristics}}{\rm{.}}$$

$${{\rm{X}}}_{1}=({{\rm{x}}}_{1}(1),\,{{\rm{x}}}_{1}(2),\ldots ,{{\rm{x}}}_{1}({\rm{n}})),$$

$${{\rm{X}}}_{2}=({{\rm{x}}}_{2}(1),{{\rm{x}}}_{2}(2),\ldots ,{{\rm{x}}}_{2}(n)),$$

$${{\rm{X}}}_{{\rm{i}}}=({{\rm{x}}}_{{\rm{i}}}(1),{{\rm{x}}}_{{\rm{i}}}(2),\ldots ,{{\rm{x}}}_{{\rm{i}}}({\rm{n}})),$$

as the sequence of system associated factors.

The correlation coefficient was defined as follows:

3 $$\xi ({x}_{o}(k),{x}_{i}(k)=\frac{\mathop{\min }\limits_{i}\,\mathop{\min }\limits_{k}|{x}_{o}(k),{x}_{i}(k)|\rho \mathop{\max }\limits_{i}\mathop{\max }\limits_{k}|{x}_{o}(k),{x}_{i}(k)|}{|{x}_{o}(k),{x}_{i}(k)|\rho \mathop{\max }\limits_{i}\mathop{\max }\limits_{k}|{x}_{o}(k),{x}_{i}(k)|}$$

The GCD was formulated as follows:

4 $$\xi ({X}_{o},X{x}_{i})=\frac{1}{n}\mathop{\sum }\limits_{k=1}^{n}\xi ({x}_{o}(k),{x}_{i}(k))$$

where, *ρ* was the distinctive coefficient lying between 0 < *ρ* < 1, and was set at 0.5.

The higher the value of the GCD, the closer the sequence of associated factors to the behavior characteristics was.

In the present study, the efficacy data (OD) was determined as the sequence of system behavioral characteristics, whereas the actual dosage of each peak (Peaks A to J) in each sample was set as the sequence of associated factors. Before data operation, normalization should be implemented on all raw data to eliminate any discrepancies based on inconformity units. The specific procedure was that each value was divided by the average of the sequence to obtain normalized results. After normalization, the data were processed using the above formulas.

#### GCA of each sample peak area with irritation

The analysis method was the same as that shown in sub-section 4.7.1, with the sequence of the system behavior characteristics changed to the ear swelling ratio.

### Artificial neural network (ANN)

The ANN is a group of models inspired by the biological central nervous system to achieve estimation functions^22^. ANN is a useful and cost-effective tool to solve complex tasks^23,24^ (Alibakshi, 2018; Leśniak and Juszczyk, 2018; Shi *et al*., 2018). At present, the most popularly used ANN is the BP neural network^25^, which is composed of the input layer, hidden layer, and output layer^26^ (Wang *et al*., 2017). It has been widely applied to functional approximation, pattern recognition, classification, and data compression^27^; however, its application for the predication and optimization of drug development, especially for multicomponent herbal medicine, is limited.

In the present study, the BP neural network was utilized to optimize the SAI components and their proportions.

#### ANN construct^28,29^

**Figure 8:**
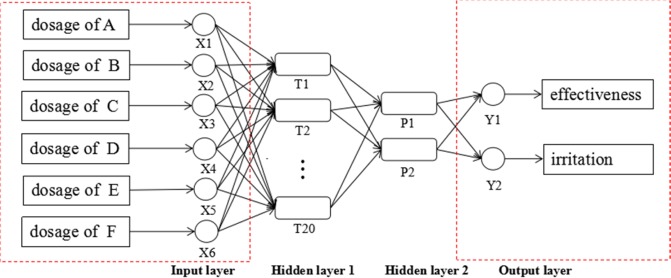
BP neural network model for optimization proportion.

The neural network toolbox of MATLAB 2016a software was applied, with the actual dosage of components in each injection sample (shown in Table 4) used as the input neuron and the absorbance and ear swelling ratios applied as the output neuron. Among these two hidden-layers were arranged the 20 and 2 neurons, respectively, to establish a four-layer structure of BP neural network jointly (shown in Fig. 8). The network was trained 1000 times and the computing precision was set at 0.0001 to determine the combination with the smallest output.

The actual dosage of each peak and the corresponding absorbance with the ear swelling ratio data of nine samples (except for sample S5) were used as the training samples of the neural network, and the gradient descent method was applied to train the neural network. The remaining sample (S5) was used as the verification sample, to evaluate the validity of the trained BP neural network.

#### ANN prediction

Based on the results of the GCA and the original ingredient proportions of the SAI products, the expected proportional range of components was set in a probable range, the output value of absorbance and ear swelling ratio was set at less than that of the NC group for the criteria, and the established BP network was operated multiple times to obtain the prospective proportion. A total of 50 groups of data that met the criteria were collected and the median proportion of each ingredient was calculated as the optimization proportion. The optimization proportion was then confirmed by importing into the established BP network again, to obtain the anticipative absorbance and ear swelling ratio data and determine whether it met the criteria or not.

### Verification of the proportion optimization

To verify the effect of the proportion optimization, we performed a validation experiment of irritation test based on the method described in section 4.5 and an anti-inflammatory efficacy test as described in section 4.6. 45 Mice were randomly divided into nine groups, 5 mice in each group: NC group, CHHU (CHHU group), A (aescinate A group), B (aescinate B group), C (aescinate C group), D (aescinate D group), E (aescinate E group), F (aescinate F group), and PO (proportion optimization group). The dosage for each group was 12.50 mg·kg^−1^.

### Declaration of interests

The authors declare that they have no known competing financial interests and non-financial interests or personal relationships that could have appeared to influence the work reported in this paper.
